# Ethical considerations in conducting surgical research in severe complicated intra-abdominal sepsis

**DOI:** 10.1186/s13017-019-0259-9

**Published:** 2019-08-05

**Authors:** Christopher J. Doig, Stacey A Page, Jessica L. McKee, Ernest E. Moore, Fikri M. Abu-Zidan, Rosemary Carroll, John C. Marshall, Peter D Faris, Matti Tolonen, Fausto Catena, Federico Cocolini, Massimo Sartelli, Luca Ansaloni, Sam F. Minor, Bruno M. Peirera, Jose J Diaz, Andrew W. Kirkpatrick, the Closed Or Open after Laparotomy (COOL) after Source Control for Severe Complicated Intra-Abdominal Sepsis Investigators, Derek J. Roberts, Ari Leppaniemi, Craig N. Jenne, Osvaldo Chiara, Paul Kubes, Braden Manns, Yoram Kluger, Gustavo P. Fraga, Bruno M. Pereira, Michael Sugrue, Teresa Holm, Jianan Ren, Chad G. Ball, Raul Coimbra, Zsolt J. Balogh, Elijah Dixon, Walter Biffl, Anthony MacLean, Ian Ball, John W. Drover, Paul B. McBeth, Juan G. Posadas-Calleja, Neil G. Parry, Salomone Di Saverio, Carlos A. Ordonez, Cino Bendinelli, Bradeon MacDonald, Michael Dunham, Artan Reso, Kelly N. Vogt, Annika Reintam Blaser, Manu Malbrain, Dario Tartaglia, Jan De Waele, Vincent Dubuisson, Hanna Lampela, Zsolt Bodnar, Arda Isik, Edoardo Picetti, Morad Hameed, Naisan R. Garraway, Lisa Julien, Sandy Widder, Norie L. Bradley, Paul T. Engels, W. Robert Leeper, Andrew Beckett

**Affiliations:** 1 Department of Critical Care Medicine, Cumming School of Medicine, University of Calgary, Calgary, Canada; 2 Department of Community Health Sciences, Cumming School of Medicine, University of Calgary, Calgary, Canada; 3 Regional Trauma Services, Foothills Medical Centre, Calgary, Canada; 4 University of Colorado, Denver, CO USA; 5 Research Facilitation Analytics (DIMR), University of Calgary, Calgary, Alberta Canada; 6 Li Ka Shing Knowledge Institute, St. Michael’s Hospital, University of Toronto, Toronto, Canada; 7 Department of Surgery, College of Medicine and Health Sciences, UAE University, Al-Ain, UAE; 8 Surgical Services John Hunter Hospital, Newcastle, NSW Australia; 9 Department of Abdominal Surgery, Abdominal Center, University of Helsinki and Helsinki University Central Hospital, Helsinki, Finland; 10 Emergency Surgery Department, Parma University Hospital, Parma, Italy; 11 General, Emergency and Trauma Surgery dept, Bufalini Hospital, Cesena, Italy; 12 Department of Surgery, Macerata Hospital, Macerata, Italy; 13 Unit of General and Emergency Surgery, Bufalini Hospital of Cesena, Cesena, Italy; 14 Department of Critical Care and Department of Surgery, NSHA- Queen Elizabeth II Health Sciences Centre, 1276 South Park Street, Halifax, Nova Scotia B3H 2Y9 Canada; 15 Division of Trauma Surgery, University of Campinas, Campinas, SP Brazil; 16 Department of Surgery, Acute Care Surgery, R Adams Cowley Shock Trauma Center, University of Maryland School on Medicine, Baltimore, MD USA; 17 Department of Critical Care Medicine, University of Calgary, Calgary, Alberta Canada; 18 Department of Surgery, University of Calgary, Calgary, Alberta Canada; 19 EG23 Foothills Medical Centre, Calgary, Alberta T2N 2 T9 Canada

**Keywords:** Intra-peritoneal sepsis, Open-abdomen, Randomized controlled trial, Multiple organ dysfunction, Consent, Waiver

## Abstract

**Background:**

Severe complicated intra-abdominal sepsis (SCIAS) has high mortality, thought due in part to progressive bio-mediator generation, systemic inflammation, and multiple organ failure. Treatment includes early antibiotics and operative source control. At surgery, open abdomen management with negative-peritoneal-pressure therapy (NPPT) has been hypothesized to mitigate MOF and death, although clinical equipoise for this operative approach exists. The Closed or Open after Laparotomy (COOL) study (https://clinicaltrials.gov/ct2/show/NCT03163095) will prospectively randomize eligible patients intra-operatively to formal abdominal closure or OA with NPTT. We review the ethical basis for conducting research in SCIAS.

**Main body:**

Research in critically ill incapacitated patients is important to advance care. Conducting research among SCIAS is complicated due to the severity of illness including delirium, need for emergent interventions, diagnostic criteria confirmed only at laparotomy, and obtundation from anaesthesia. In other circumstances involving critically ill patients, clinical experts have worked closely with ethicists to apply principles that balance the rights of patients whilst simultaneously permitting inclusion in research. In Canada, the Tri-Council Policy Statement-2 (TCPS-2) describes six criteria that permit study enrollment and randomization in such situations: (a) serious threat to the prospective participant requires immediate intervention; (b) either no standard efficacious care exists or the research offers realistic possibility of direct benefit; (c) risks are not greater than that involved in standard care or are clearly justified by prospect for direct benefits; (d) prospective participant is unconscious or lacks capacity to understand the complexities of the research; (e) third-party authorization cannot be secured in sufficient time; and (f) no relevant prior directives are known to exist that preclude participation. TCPS-2 criteria are in principle not dissimilar to other (inter)national criteria. The COOL study will use waiver of consent to initiate enrollment and randomization, followed by surrogate or proxy consent, and finally delayed informed consent in subjects that survive and regain capacity.

**Conclusions:**

A delayed consent mechanism is a practical and ethical solution to challenges in research in SCIAS. The ultimate goal of consent is to balance respect for patient participants and to permit participation in new trials with a reasonable opportunity for improved outcome and minimal risk of harm.

## Introduction

Patients with severe sepsis and multiple organ dysfunction have a high mortality rate; their care is expensive [1, 2]. Sepsis is a common cause of death worldwide [3, 4], with an increasing incidence estimated at between 18 and 31 million cases worldwide per year [4–8]. Mortality approaches 30–40% when shock is present [9–11], and is higher in countries without advanced acute care hospitals with fully resourced intensive care units [3]. Sepsis management is a tremendous burden to society; in the USA, it ranked highest among admissions for all disease states, accounted for more than $24 billion in hospital expenses in 2013, represented 13% of total hospital costs, yet accounted for only 3.6% of hospital length of stays [12, 13].

Severe complicated intra-abdominal sepsis (SCIAS) is a particular challenge as early surgical source control should be part of initial therapy [14]. Because of progressive inflammation, SCIAS frequently progresses to septic shock, multiple organ dysfunction, and often death [15]. Inflammation associated with intra-abdominal sepsis may result in significant ‘third-spacing’ of fluid, and development of raised abdominal pressure further affecting cardiopulmonary and renal function. The commonly accepted surgical approach at completion of a (source control) laparotomy is to close the abdominal wall fascia in a manner similar to any other surgical procedure without contemplating the unique biological and inflammatory mechanisms in SCIAS and the consequences of intra-abdominal hypertension. An alternate surgical approach for SCIAS is to leave the abdominal cavity ‘open’, applying (through various techniques) negative pressure therapy within the peritoneal cavity [16]. The ‘open’ surgical approach has been widely adopted based on basic science data which suggests this prevents dysregulated inflammation, encouraging but preliminary human studies [17–19]. Both approaches are used, and each approach may have unique benefits and risks. There is a lack of consensus and equipoise on which approach is more efficacious. There is a need for methodologically rigorous clinical trials to compare ‘open’ versus ‘closed’ surgical management [20].

The Closed or Open after Laparotomy (COOL) study is a multinational randomized controlled clinical trial comparing an ‘open abdomen’ (OA) approach to closed surgical management (https://clinicaltrials.gov/ct2/show/NCT03163095). The University of Calgary is both the sponsoring institution and the pilot centre. The original ethics submission was made to the Conjoint Health Research Ethics Board of the University of Calgary: the CHREB must follow all human research regulations under Canadian Law and The Tri-Council Policy Statement-2. It also complies with The International Conference for Harmonisation (ICH) Guidance E6: the Good Clinical Practice (GCP) guideline. However, the COOL trial is an international collaboration involving investigators, medical centres, and medicolegal systems across many different countries. Recognizing that there is a gross global imbalance in the funding directed to a disease that affects patients irrespective of country/health system [21, 22], the COOL trial will attempt to encourage global participation. Thus, this review will discuss the relevant principles of performing emergency research in critically ill surgical patients in general and as they specifically apply to the COOL study.

## Research ethics and informed consent

The necessity of informed consent for participation in research arises from one of the darkest parts of modern medical profession’s history, and also one of its most enlightening. In the early to mid-twentieth century, there were egregious examples of research involving humans where informed consent was not obtained and individuals suffered serious and significant harm including death [23, 24]. Attention has rightly been focused on the horrors of concentration camps operated by the Nazi regime in Germany and the occupied countries. In these concentration camps were repeated examples of experimentation on human subjects including research on hypothermia, extreme starvation, wound management, and eugenics. Following the Second World War, the trials of Nazi medical personnel at Nuremburg informed the world of some of these atrocities and resulted in the Nuremburg Code [23, 24]. The first of the 10 points of the Nuremburg Code stated ‘Required is the voluntary, well-informed, understanding consent of the human subject in a full legal capacity.’ The remaining nine points identified principles that are also important (and relevant for surgical research) including a biological basis that justifies the research, minimizing risk, a balance of risk and benefit, protection of participant’s well-being, a necessity to stop an individual’s participation if unduly dangerous, and the requirement to permit a subject’s withdrawal of consent [23, 25, 26]. In 1964, The World Medical Association adopted the Declaration of Helsinki, which emphasized the fundamental right of self-determination practically manifest as consent being the right to make informed decisions [25, 27, 28]. The Declaration also identified that informed consent may be provided by a person other than the subject if the subject themselves was not capable [28].

Despite the Code and the Declaration, ongoing evidence of research malfeasance continued. In the late 1920s, sharecroppers in the USA were recruited for a study to examine the natural history of syphilis (Tuskegee Syphilis Study): this study continued until it was exposed in the media in the late 1960s [23]. In 1966, Henry Beecher published a case series of clinical research studies with glaring violations of appropriate conduct. He concluded ‘what seem to be breaches of ethical conduct in experimentation are by no means rare…examples could be easily found…’ [23]. Arising from Beecher’s report, and other examples such as the Tuskegee Syphilis study, the National Commission for the Protection of Human Subjects of Biomedical and Behavioral Research met, and in 1978 issued the ‘Belmont Report: Ethical Principles and Guidelines for the Protection of Human Subjects of Research.’ [29]. The Belmont Report emphasized 3 key aspects of research ethics: respect for persons (autonomy), beneficence (maximizing benefits and minimizing harms), and justice (fair distribution of risks/benefits/costs in a non-exploitive manner). The application of these principles emphasized the selection of subjects, a risk-benefit assessment, and informed consent [29, 30]. Critiques of the Belmont Report include that the 3 principles are not presented in a ‘weighted’ manner, i.e. that a principle such as respect for persons should have primacy [31]. In response, at least one member of the panel, Dr. Al Jonsen, stated that it is the responsibility of individual (investigators) and institution review boards to evaluate each research proposal uniquely and apply the principles appropriate to the proposed research [32]. The Nuremburg Code, the Declaration of Helsinki, and the Belmont Report form a historical basis for current national and international research standards.

For example, The International Conference for Harmonization (ICH) Guidance E6: the Good Clinical Practice (GCP) Consolidated Guidelines [33] has its roots in the Declaration of Helsinki and focuses on the protection of research subjects and the credibility and validity of research findings. It is an accepted standard in many countries (such as Canada, the European Union, Japan, Australia, and the USA) as a definitive quality standard of conducting clinical trials for pharmaceutical research. ICH GCP guidelines include expectations of institutional research boards/research ethics boards related to board composition, protocol review, and processes related to consent. Although ICH GCP guidelines only pertain to pharmaceuticals and these guidelines are not necessarily part of the regulations of individual countries, the GCP has become a widely adopted pragmatic standard. Institutions, such as hospitals or universities with medical schools, where pharmaceutical research is conducted have IRB’s/REB’s which must adhere to the principles of the guidelines; as such, they have become in many ways de facto applied to most human-based clinical research. This GCP includes provisions on processes when consent cannot be obtained from study participants or their legally authorized representatives (E6 (R2) 4.8.15).

Research funded by US government agencies are governed by ‘The Common Rule’ or the Department of Health and Human Services ‘Federal Policy for the Protection of Human Participants (45 CFR 46, Subpart A)’. The Belmont Report serves as the basis for The Common Rule which defines the basic principles of research ethics involving human participants. In 2001, Canada’s three federal research agencies, the Canadian Institute for Health Research (CIHR), the Natural Sciences and Engineering Research Council of Canada (NSERC), and the Social Sciences and Humanities Research Council (SSHRC), jointly created the Interagency Advisory Panel on Research Ethics (PRE or the Panel) as part of a collaborative effort to promote the ethical conduct of research involving human participants [34]. The Advisory Panel has published the Tri-Council Policy Statement-2 which provides requirements for institutions who conduct human-based research and receive funding from one of these agencies.

All of these guidelines and policies emphasize, in keeping with the principle of Respect for Persons, that at enrolment, participant consent is expected as a normative standard. However, these also recognize and support that there are exceptions to this general ethical requirement which apply in specific situations such as emergency research [34, 35]. Across all of these guidelines or regulations is an acceptance that research should be inclusive and that research in emergency situations may provide life-saving benefits, and therefore, consent processes have to be developed and implemented to permit research in emergency setting where consent cannot be obtained. In emergency research, potential participants may not have the capacity to provide informed consent at enrolment. Under these circumstances, patients are considered particularly vulnerable and are owed special ethical obligations and protection commensurate with the risks involved. The research participant’s welfare should be protected by additional safeguards, where feasible and appropriate [34].

## Clinical equipoise

A major ethical tenet underlying all clinical research but in particular randomized clinical trials has been the concept of clinical equipoise perhaps best classically formulated by Benjamin Freedman. Freedman defined equipoise as ‘genuine uncertainty within the expert medical community—not necessarily on the part of the individual investigator—about the preferred treatment.’ Although many criticisms have been put forward as to the appropriateness of equipoise as a sole justification for proceeding with a clinical trial, once a decision has been made on a broader context to proceed with a clinical trial, clinical equipoise maintains import in that it helps to sharpen focus on whether study treatment arms are reasonably comparable. Furthermore, equipoise places emphasis on informing patients on the honest disagreement among expert clinicians as to which therapy is proven or a matter of professional preference [36]. If both are considered reasonably comparable and, particularly, if both are already commonly used (i.e. a trial of 2 accepted forms of [surgical] therapy) then equipoise is also relevant for considering the ethical propriety of waiver or deferral of consent. Criticisms with this principle include that it is often narrowly interpreted within only a clinical context, rather than in a broader societal interest in evidence-based policy. Gamble commented on the irony that informed consent is not required for treatment with non-validated therapies that are currently in practice but for which there is a lack of evidence in the benefits and risks [37].

## The exigency pressing necessity for high quality surgical research

In general, the overall quality of surgical research has been criticized as being grossly inadequate to properly guide scientifically informed decision making despite the importance these decisions have in determining whether patients die or are permanently impaired from surgical emergencies [38, 39]. One famous commentary compared surgical research to ‘comic opera’ [40], lamenting the reliance on retrospective case series as a methodology, and another referred to the typical retrospective case series as ‘research waste’ [38]. However, it has been countered that surgical research is difficult to properly conduct as there are so many practical barriers to conducting RCTs especially in urgent life-threatening situations [41], and thus, RCTs make up only a small proportion of published surgical research [38, 39]. Retrospective case series predominate, potentially because they are vastly easier to conduct and are free of regulatory hurdles that accompany conducting an RCT, but are still publishable in journals and offer career advancement to investigators. Further, RCTs are not required by device manufacturers or regulators to allow market entry [38]. Unfortunately, medical history is replete with examples of where non-randomized, biased research provided misinformation that led to worse clinical outcomes, because the more difficult but required RCTs were not performed [42, 43].

## Consent processes for SCIAS and the COOL trial in Canada

The COOL trial will be conducted across the globe and thus will involve multiple countries, hospitals, cultures, and medicolegal systems. The COOL investigators assessed that a priori informed consent from potential participants was impractical because (1) inclusion criteria could only be identified in the operating room, (2) there was not a reasonable way to identify potential participants preoperatively and reviewing ‘possible’ enrolment could reasonably be confused/conflated with consent for the clinical operative consent, and (3) consent within the operating room could not feasibly be obtained from a legally authorized representative without the risk for an unacceptable significant delay in a potentially unstable patient. Therefore, a decision was made to seek approval from our CHREB to use delayed or deferred consent as the COOL trial met the alterations on consent requirements described in article 3.8a of the TCPS2 (Table 1) [34].

**Table 1:** Required Criteria for Medical Emergencies carried out without the consent of participants

a. A serious threat to the prospective participant requires immediate intervention;	
b. Either no standard efficacious care exists or the research offers a realistic possibility of direct benefit to the participant in comparison with standard care;	
c. Either the risk is not greater than that involved in standard efficacious care, or it is clearly justified by the prospect for direct benefits to the participant;	
d. The prospective participant is unconscious or lacks capacity to understand the risks, methods and purposes of the research project;	
e. Third-party authorization cannot be secured in sufficient time, despite diligent and documented efforts to do so; and	
f. No relevant prior directive by the participant is known to exist.	

### Requirement 1

A serious threat to the prospective participant requires immediate intervention;

Sepsis is unpredictable and deadly with sudden onset, and intra-abdominal sepsis is one of its most complicated forms [44–46]. Mortality in SCIAS approaches 30–40% when shock is present [9, 11], and this may be 80% in the developing world [3]. Once identified, such patients require immediate surgical intervention. The failure to promptly obtain adequate source control has been described as an independent predictor of mortality in those with this condition [47]. Thus, any delay in treatment, even to ensure informed consent, could slow the patients care and negatively impact their care and outcomes. Similar to the hemorrhaging patient, the septic patient needs immediate management and it is because of the urgent requirement for treatment that it is impossible or impracticable to obtain consent prior to treatment.

### Requirement 2

No standard efficacious care exists or the research offers a realistic possibility of direct benefit to the participant in comparison with standard care;

### Requirement 3

Risk is not greater than that involved in standard efficacious care, or it is clearly justified by the prospect for direct benefits to the participant;

The COOL study compares two operative management strategies both of which are considered a standard of care for SCIAS; as such, either treatment allocation may carry unique risks or benefits, but that there is an equipoise as to a preferred management approach [48, 49]. Neither of the treatment allocation arms, open or closed management, are new or novel. Despite being of more recent interest, the use of the OA technique historically dates back at least 75 years [50]. The closed management strategy is the common approach for the vast majority of elective and urgent abdominal surgical procedures. Its adoption for SCIAS has not considered the unique inflammatory consequences of this condition, including the development of intra-abdominal hypertension/abdominal compartment syndrome, and the contribution of undrained inflammatory mediators to the development of organ dysfunction. Thus, two well established and currently utilized standards of care for managing the abdominal cavity after source control laparotomies are being compared, recognizing that all patients will be undergoing source control laparotomies. Neither method is considered more efficacious, and multiple extensive reviews of the existing world literature cannot provide any further guidance beyond biased opinion [44, 48, 51–53]. Both approaches offer known and realistic possible risks to the patient. It is important to note that this patient population will be very sick, and either therapy can be associated with dangerous complications during the surgical treatment of abdominal sepsis.

### Requirement 4

The prospective participant is unconscious or lacks capacity to understand the risks, methods and purposes of the research project;

Any patient being considered for enrollment will only meet inclusion criteria during the operation: by definition, they will be under a general anaesthetic and unable to provide consent. Preoperatively, most of these patients will also be very ill and, through the nature of severe sepsis, have diminished mental capacity due to multi-system organ dysfunction [9, 54, 55]. Sepsis is often accompanied by an acute encephalopathy which, when present, not only worsens prognosis but grossly impairs normal information processing [56, 57]. Various patterns of brain imaging findings have been described in adult patients with acute sepsis and include cytotoxic edema, vasogenic edema, posterior reversible encephalopathy syndrome, white matter disruption, and brain atrophy [54, 58, 59]. Thus, those with SCIAS will have many threats to the normal cognition, which will typically require a long period of postoperative convalescence prior to full neurocognitive recovery. Most patients with preoperative ‘acute abdomens’ will not meet inclusion criteria intra-operatively (as SCIAS, and the inclusion criteria of COOL, specifically define a narrow subset of patients with intra-abdominal sepsis and evidence of shock/multi-organ dysfunction). Therefore, preoperatively identifying patients, and approaching legally authorized representatives, are not feasible.

### Requirement 5

Third-party authorization cannot be secured in sufficient time, despite diligent and documented efforts to do so

True study eligibility for COOL will only be finally ascertained once the patient is in the operating room with an open peritoneal cavity and the true extent of intra-peritoneal contamination is appreciated [51]. Due to the urgent nature of the treatment required in this population, attempting to obtain surrogate or third-party authorization would greatly slow patient care and treatment and would certainly prolong the duration of general anesthetic. Research discussed further below also supports the contention that a delayed consent paradigm provides surrogates with the ability to understand trial information better than when presented in critical stressful time-pressured situations [57, 60].

### Requirement 6

No relevant prior directive by the participant is known to exist

If such a directive is known, the patient would not be included in the study. This factor may be more pertinent in jurisdictions that consider community consultation as desirable. Unfortunately, SCIAS can afflict any individual regardless of underlying health, making it difficult to identify any particular sub-populations of increased risk.

**Fig. 1:**
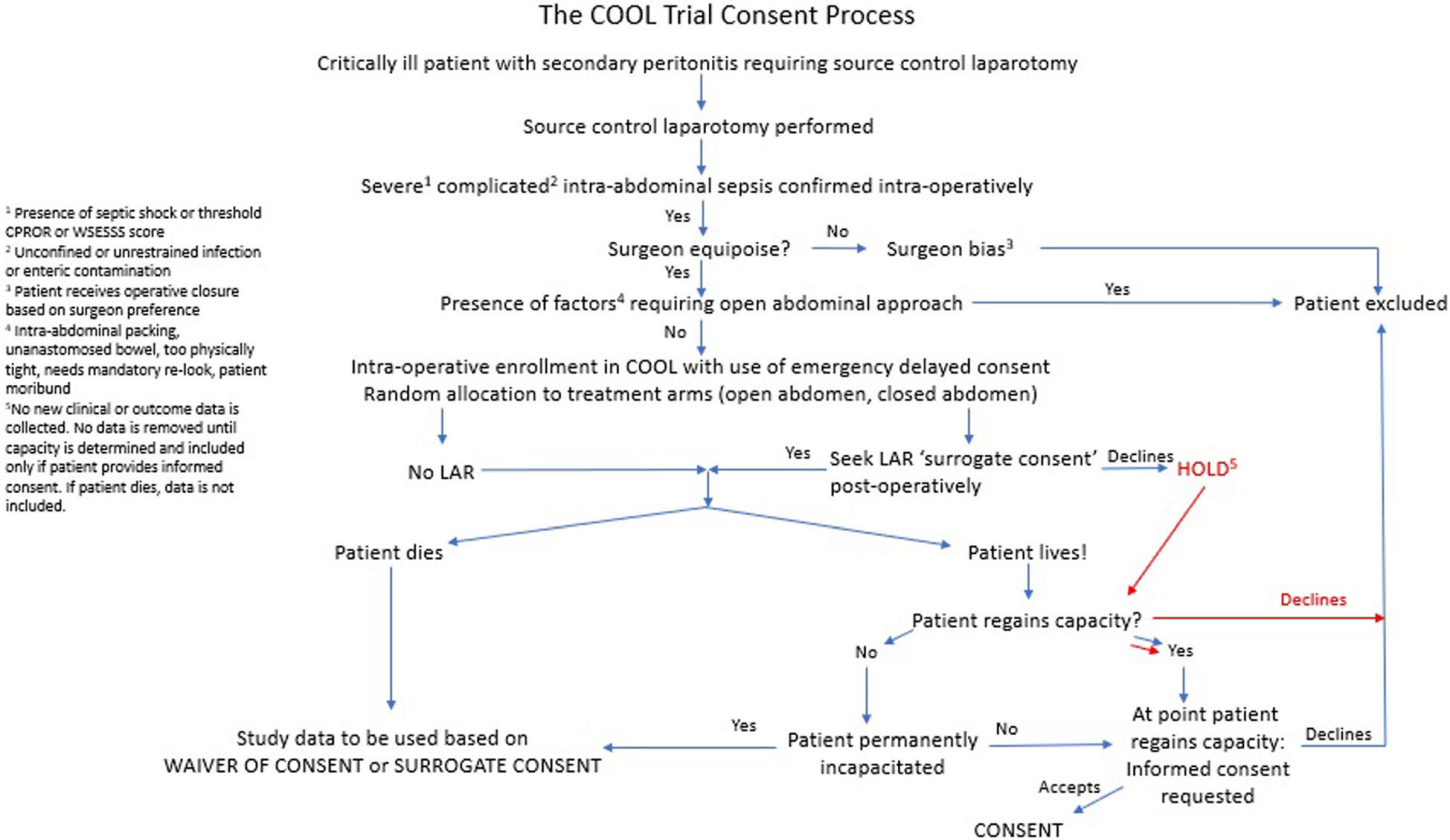
Operationalization of the COOL Trial Informed Consent Process

Therefore, emergency consent appears both justified and required in order to be able to conduct COOL and to address the research question properly (Fig. 1).

## Discussion

All human research must conform to basic principles of integrity and respect for human dignity. Specific approaches and regulatory details have varied somewhat around the world with different national or regional systems attempting to appropriately balance the competing requirement of adequate prospective informed consent against the benefits of conducting challenging clinical research [37, 61]. Largent stressed that the critical philosophical values, typically secured through informed consent, of respect for patient autonomy and protection of patient well-being, can still be secured to proceed with emergency research without initial consent when certain conditions are met [61]. These conditions are (1) responsiveness (the experimental intervention must be responsive to an urgent medical need of the patients), (2) comparable risk-benefit ratio (the risk-benefit ratio of the experimental intervention is favorable, and at least as favorable as that of available alternatives and the control, if any), (3) no conflicting preferences (there is no compelling reason to think that participation in the research conflicts with enrolled patients’ values or interests), (4) minimal net risks (nonbeneficial procedures included in the study cumulatively pose no greater than minimal risk), and (5) prompt consent (consent for ongoing and additional emergency research interventions is obtained as soon as possible) [61].

Truog and colleagues [62] recommended, applied narrowly and conservatively, that a patient’s general consent for treatment should serve as authorization for participation in a clinical trial without seeking separate [a priori] specific consent if:

1. All the treatments in the trial are offered outside the trial: a trial comparing 2 therapies that are already in use.
2. The treatments within each arm of the study involve similar risks to each other, and not greater risks compared to other reasonable treatment alternatives.
3. Clinical equipoise must exist between treatments.
4. No reasonable person should have a preference for one treatment over another, and this should cover direct and indirect effects of the treatment. The ‘reasonable-person’ standard is best applied by the local research ethics board/institution review board.
5. Patients should be informed that the institution uses this standard.

For the purposes of the COOL study, the first 4 of the above 5 criterion are met. The fifth criterion is perhaps beyond the scope of an individual clinical trial, but perhaps the COOL study provides the opportunity for participating centres to consider what the standard for research under similar circumstances should be. Truog provides examples of research that might be relevant: for example, 2 approved antibiotics for preoperative prophylaxis, whether low-dose anticoagulants improves longevity of intravascular catheters, a study to determine speed to resumption of spontaneous unassisted breathing [weaning] in ventilated patients, or in general studies that fall under the heading of quality improvement [62].

A deferred consent process allows initial enrollment of incapacitated patients into an approved clinical trial with the expectation that a valid informed consent will be obtained when the patient regains capacity and can fully understand and appreciate the details of the proposed research. The enrollment and treatment allocation cannot be ‘un-done’ but the patient or their legal representatives can thereafter decline further participation and may have their data and any biological samples destroyed. This potential enrollment process recognizes that there is continual tension in balancing the requirements for informed consent and the need to advance knowledge regarding critical medical conditions with a high mortality rate and treatment uncertainties [37, 63, 64].

Waiving the need for immediate consent and deferring consent to the postoperative period avoids delaying or prolonging emergency interventions whilst ensuring permission for ongoing study participation and use of data. Many countries around the world including the USA, Canada, Australia, the UK, and the European Union permit deferred consent [61, 63]. For instance, a deferred consent process was used in both the land-mark SAFE trial involving nearly 7000 critically ill patients in 16 academic tertiary hospitals in Australia and New Zealand [65], and in the CRASH-II trial of 20211 injured adults in 274 hospitals in 40 countries [66]. In the UK, a deferred consent process for children has been in place since 2008, provided that treatment is urgently needed, urgent action is needed for the purposes of the trial, it is not reasonably practicable to obtain consent prospectively, and an ethics committee has given approval to the procedure under which the action is taken [37, 67]. Deferred consent is distinct from surrogate consent, or consent from a legally authorized representative. There are concerns regarding the validity of surrogate or LAR consent in urgent, stressful situations such as clinical emergencies [37, 68]. Some populations such as the socioeconomically disadvantaged often lack surrogates. Therefore, relying on surrogate consent may introduce selection bias and threaten the internal validity of the research findings [61].

Although such consent processes are quite new, the limited study of these methods themselves has been favorable. Gamble and colleagues found there was a higher rate for emergency as compared with elective enrollments, which they interpreted to suggest that there may be a greater capacity for informed decision making when parents of critically ill patients were approached after a critical medical emergency, in a potentially less stressful environment [37]. Woolfall also noted that when surveyed after the deferred consent process both parents and practitioners supported the use of deferred consent, both in the trial they were involved with and its potential use in future trials [63]. They did caution, however, that surrogates were very sensitive to the timing of the deferred consent process [63], which speaks to the need to consider the potential neurocognitive recovery of COOL patients, or potential lack thereof [69]. Their work also revealed that delayed consent allowed surrogates to express a sense that they could understand trial information better than if it was presented earlier when the context was more stressful, findings applicable to patients themselves faced with potential decision making in critical illness [63]. However, it should be noted that public acceptance of the deferred consent process has not been universally consistent, and further work is justified to understand the patient and legally authorized representative’s perspectives on these issues is warranted [70]. It is possible that there may be disconnect between how respondents felt about deferred consent in theory and how they perceived the process in the real world [70]. Therefore, all efforts should be made to refine the consent process in surgical emergencies to both enhance patient and family comfort but to also allow studies of potentially life-saving interventions to practically proceed.

It has been stated that ethical practice requires that there must be a state of clinical equipoise regarding the merits of the two strategies to be tested, and the trial must be designed in such a way to make it reasonable to expect that if the trial is successfully concluded, clinical equipoise would be disturbed and the results convincing enough to provide a clear answer to clinicians [36]. With the increasing, but potentially unwarranted recommendations to utilize the open abdomen in SCIAS [16, 71], there is somewhat of an urgency to conduct an appropriate trial. In contrast to more formalized and recognized approaches to pharmaceutical development, advances in surgery are often unregulated, unstructured, and variable. The IDEAL model, however, has attempted to delineate general stages of surgical development as constituting the stages of innovation, development, exploration, assessment, and long-term study [38, 72]. Use of the open abdomen for SCIAS is currently in the stages of Exploration and Assessment according to the IDEAL model [38, 72], depending on the local practices of an institution. If the opportunity for robust evaluation of open abdomen is not seized, widespread adoption of this technique may occur without adequate evidence of efficacy making future study impossible [38, 41].

Due to the fact that the COOL trial will compare two standards of care that have complete equipoise in the surgical community and there is an urgent requirement for treatment in this patient population, delayed consent is the safest and only practical way to answer the question about which method is the best practice, without having a negative impact on patient care. The COOL trial is currently approved by the Conjoint Research Ethics Board of the University of Calgary (REB-16-1588) to proceed with a delayed consent process given the time-sensitive critical nature of decision making. Research ethics will vary throughout the world, and it is anticipated that various local policies concerning community consent, waiver of consent, or informed consent of significant patient proxies will vary among the local approaches to ensure the COOL trial is performed to the what is perceived to be the highest ethics standard in each participating jurisdiction. All participating Institutions will thus be required to obtain research ethics certification (approval). This paradigm will involve the highest standards, formally recognizing that the COOL study will be conducted in accordance with Good Clinical Practice Guidelines and applicable regulatory requirements in all health care systems at all times.

The COOL investigators recognize an obligation to patients with SCIAS to provide the best care and to conduct ethical research. The Hippocratic Oath requires physicians to ‘consider for the benefit of my patients and abstain from whatever is deleterious and mischievous’ and to ‘give no deadly medicine to any one if asked, nor suggest any such counsel’. It has been emphasized that competent and ethical medicine is social rather than individual in nature. Thus, progress relies on progressive consensus within the medical community [36]. However consensus opinions based on anything less than publicly presented evidence should only be used to guide patient treatment not hunches or guides or personal preferences based on anything less [36].

## Conclusions

A delayed consent mechanism is a practical and ethical solution to challenges in research in SCIAS. The ultimate goal of consent is to balance respect for patient participants and to permit participation in new and urgently needed trials such as COOL with a reasonable opportunity for improved outcome and minimal risk of harm.

## Data Availability

Not applicable
