# Correction to: Ethical considerations in conducting surgical research in severe complicated intra-abdominal sepsis

**DOI:** 10.1186/s13017-019-0268-8

**Published:** 2019-10-17

**Authors:** Christopher J. Doig, Stacey A. Page, Jessica L. McKee, Ernest E. Moore, Fikri M. Abu-Zidan, Rosemary Carroll, John C. Marshall, Peter D. Faris, Matti Tolonen, Fausto Catena, Federico Coccolini, Massimo Sartelli, Luca Ansaloni, Sam F. Minor, Bruno M. Peirera, Jose J. Diaz, Andrew W. Kirkpatrick, Closed Or Open after Laparotomy (COOL) after Source Control for Severe Complicated Intra-Abdominal Sepsis Investigators

**Affiliations:** 1 Department of Critical Care Medicine, Cumming School of Medicine, University of Calgary, Calgary, Canada; 2 Department of Community Health Sciences, Cumming School of Medicine, University of Calgary, Calgary, Canada; 3 Regional Trauma Services, Foothills Medical Centre, Calgary, Canada; 4 University of Colorado, Denver, CO USA; 5 Research Facilitation Analytics (DIMR), University of Calgary, Calgary, Alberta Canada; 6 Li Ka Shing Knowledge Institute, St. Michael’s Hospital, University of Toronto, Toronto, Canada; 7 Department of Surgery, College of Medicine and Health Sciences, UAE University, Al-Ain, UAE; 8 Surgical Services John Hunter Hospital, Newcastle, NSW Australia; 9 Department of Abdominal Surgery, Abdominal Center, University of Helsinki and Helsinki University Central Hospital, Helsinki, Finland; 10 Emergency Surgery Department, Parma University Hospital, Parma, Italy; 11 General, Emergency and Trauma Surgery dept, Bufalini Hospital, Cesena, Italy; 12 Department of Surgery, Macerata Hospital, Macerata, Italy; 13 Unit of General and Emergency Surgery, Bufalini Hospital of Cesena, Cesena, Italy; 14 Department of Critical Care and Department of Surgery, NSHA- Queen Elizabeth II Health Sciences Centre, 1276 South Park Street, Halifax, Nova Scotia B3H 2Y9 Canada; 15 Division of Trauma Surgery, University of Campinas, Campinas, SP Brazil; 16 Department of Surgery, Acute Care Surgery, R Adams Cowley Shock Trauma Center, University of Maryland School on Medicine, Baltimore, MD USA; 17 Department of Critical Care Medicine, University of Calgary, Calgary, Alberta Canada; 18 Department of Surgery, University of Calgary, Calgary, Alberta Canada; 19 EG23 Foothills Medical Centre, Calgary, Alberta T2N 2 T9 Canada

**Correction to: World J Emerg Surg (2019) 14:39**

**https://doi.org/10.1186/s13017-019-0259-9**

The original article [1] contained a typo in author, Federico Coccolini’s name. This has now been corrected.

## References

[1] Doig CJ, Page SA, McKee JL, Moore EE, Abu-Zidan FM, Carroll R (2019). Ethical considerations in conducting surgical research in severe complicated intra-abdominal sepsis. World J Emerg Surg.

